# Biomineralization of lithium nanoparticles by Li-resistant *Pseudomonas rodhesiae* isolated from the Atacama salt flat

**DOI:** 10.1186/s40659-022-00382-6

**Published:** 2022-03-16

**Authors:** N. Bruna, E. Galliani, P. Oyarzún, D. Bravo, F. Fuentes, J. M. Pérez-Donoso

**Affiliations:** 1 BioNanotechnology and Microbiology Laboratory, Center for Bioinformatics and Integrative Biology (CBIB), Facultad de Ciencias de la Vida, Av. República # 330, Santiago, Chile; 2 Laboratorio de Análisis de Sólidos, Departamento de Ciencias Químicas, Facultad de Ciencias Exactas, Universidad Andrés Bello, Santiago, Chile; 3 Laboratorio de Microbiología Oral, Facultad de Odontología, Universidad de Chile, Santiago, Chile; 4 Escuela de Geología, Facultad de Ciencias, Universidad Mayor, Av. Manuel Montt 367, Santiago, Chile

**Keywords:** Nanoparticles, Biomineralization, Bionanotechnology, Atacama Desert, Lithium nanoparticles

## Abstract

**Background:**

The Atacama salt flat is located in northern Chile, at 2300 m above sea level, and has a high concentration of lithium, being one of the main extraction sites in the world. The effect of lithium on microorganism communities inhabiting environments with high concentrations of this metal has been scarcely studied. A few works have studied the microorganisms present in lithium-rich salt flats (Uyuni and Hombre Muerto in Bolivia and Argentina, respectively). Nanocrystals formation through biological mineralization has been described as an alternative for microorganisms living in metal-rich environments to cope with metal ions. However, bacterial lithium biomineralization of lithium nanostructures has not been published to date. In the present work, we studied lithium-rich soils of the Atacama salt flat and reported for the first time the biological synthesis of Li nanoparticles.

**Results:**

Bacterial communities were evaluated and a high abundance of *Cellulomonas*, *Arcticibacter*, *Mucilaginibacter*, and *Pseudomonas* were determined. Three lithium resistant strains corresponding to *Pseudomonas rodhesiae*, *Planomicrobium koreense*, and *Pseudomonas* sp. were isolated (MIC > 700 mM). High levels of S^2−^ were detected in the headspace of *P. rodhesiae* and *Pseudomonas* sp. cultures exposed to cysteine. Accordingly, biomineralization of lithium sulfide-containing nanomaterials was determined in *P. rodhesiae* exposed to lithium salts and cysteine. Transmission electron microscopy (TEM) analysis of ultrathin sections of *P. rodhesiae* cells biomineralizing lithium revealed the presence of nanometric materials. Lithium sulfide-containing nanomaterials were purified, and their size and shape determined by dynamic light scattering and TEM. Spherical nanoparticles with an average size < 40 nm and a hydrodynamic size ~ 44.62 nm were determined.

**Conclusions:**

We characterized the bacterial communities inhabiting Li-rich extreme environments and reported for the first time the biomineralization of Li-containing nanomaterials by Li-resistant bacteria. The biosynthesis method described in this report could be used to recover lithium from waste batteries and thus provide a solution to the accumulation of batteries.

**Supplementary Information:**

The online version contains supplementary material available at 10.1186/s40659-022-00382-6.

## Background

During the last decades, the different technological applications of lithium have increased the interest in their extraction and in the generation of new lithium-based materials. A main feature of lithium is its high specific heat [3582 J/(g K)] and low standard reduction potential (− 3040 V), which has allowed its use in the manufacture of lubricating greases, ceramic glasses, and rechargeable batteries [1–3]. Lithium is mainly extracted from brines to produce different salts such as lithium chloride, acetate, hydroxide, and carbonate. Lithium is obtained from minerals such as spodumene, petalite, amblygonite, and lepidolite [4, 5]. The United States Geological Survey (USGS) has estimated that world lithium reserves reach 34 million tons, with a 70% present in brines (lithium deposits in solution) [1]. According to this report, Chile possesses 7.5 million tons of lithium in brines, most of which are located in the Atacama salt flat.

The exploitation of lithium from Atacama salt flats has continuously grown during the last decade as a consequence of the increasing energy consumption and the use of lithium-ion rechargeable batteries [6–9]. The high electrochemical potential of lithium favors its use in batteries and allows the storage of large amounts of energy (energy density) [10]. In general, most lithium batteries are constituted by a LiCoO_2_ cathode, a graphite anode, and an electrolyte composed of lithium salts in organic solvents, allowing the movement of ions between the cathode and the anode [10, 11].

In recent years, new technologies for rechargeable batteries based on the use of Li–S nanomaterials at the cathode have been developed [12–16]. These batteries have a higher energy density than Li-ion batteries. However, a challenge for the development of this type of battery is controlling the synthesis of lithium sulfide nanoparticles (NPs) to avoid the formation of other lithium polysulfides [17]. The synthesis of lithium NPs is a complex and expensive process since it involves high temperatures and anaerobic conditions due to the high reactivity of lithium in presence of oxygen [12, 14].

During the last decade, the use of microorganisms as biofactories for the synthesis of different metal sulfide nanoparticles has emerged as a novel, efficient, and environmentally friendly method [18, 19]. However, the biosynthesis of lithium nanoparticles has not been reported to date. The use of bacterial cells to synthesize metal sulfide nanomaterials has been described for a number of metals, being the most common CdS, ZnS, and Ag_2_S [20–26]. In general, the biosynthesis of metal sulfide NPs requires low concentrations of the metal (a non-toxic dose), low temperatures (optimal growth temperature of the microorganism), and an external source of S, such as reduced glutathione (GSH), cysteine (Cys), or mercaptosuccinic acid (MSA) [27].

Recently, our research group reported the biosynthesis of metal sulfide NPs using extremophile microorganisms inhabiting desert environments. In particular, the biosynthesis of CdS nanoparticles using acidophilic and halophilic microorganisms was reported for the first time [20, 24, 28]. Based on this, we hypothesized that the lithium-rich zone of the Atacama Desert contains lithium-resistant bacteria with high capacity to produce sulfide, which can be used for the biosynthesis of lithium sulfide nanoparticles. This work describes the chemical and biological characterization of Atacama salt flat samples, and the isolation of the first microorganism capable of biomineralizing lithium in the form of lithium sulfide nanoparticles.

## Materials and methods

### Sampling

A surface soil sample (500 g) was obtained from the Atacama Desert in the Atacama salt flat. The sample was placed in sterile bags and transferred to the laboratory. The geographical coordinate (DMS) of the collected sample was 22º 59′ 08.11″ S, 68º 09′ 05,81″ W. The sample was stored at 4 ºC before being processed in the laboratory.

### X-ray diffraction (XRD)

The XRD assay was carried out in the Solid Analysis Laboratory (L.A.S) at Andrés Bello University. For this, the soil sample was pulverized and then micronized to a size of 5 to 10 µm. The diffractogram was obtained by the Debye–Scherrer method using a Bruker D8 Advance diffractometer, with a LynxEyer linear detector, for polycrystalline samples. A wavelength CuKα1 = 1.5406 Ă was used, with a power of 40 kV/30 mA, scanning at a speed of 0.01° 2θ every 0.5 s, with an angular measurement range from 2θ = 5° up to 2θ = 80°. Subsequently, the phases were identified with the analysis software Diffrac Suite v 25.2011 (Diffrac.EVA v2.1), which uses the Crystallography Open Database (COD, version 2011).

### Analysis of microbial communities

DNA extraction was performed using 250 mg of soil and the PowerSoil DNA Isolation Kit (Qiagen). Then, total DNA was quantified using Qubit fluorometer (Invitrogen). Obtained DNA was sequenced in the Argonne National Laboratories using the Earth Microbiome Project barcoded primer set, adapted for the Illumina HiSeq2000 and MiSeq following a previously reported protocol [29, 30]. The V4 region was amplified using primers 515F and 806R (5'GTGCCAGCMGCCGCGGTAA and 5′-GGACTACHVHHHTWTCTAAT). Sequence analysis was performed using the DADA2 bioinformatics tool under default settings (https://benjjneb.github.io/dada2/) [31]. The taxonomic assignments were made through the SILVA database (version 132) [32]. According to the quality profiles produced, the sequences were cut at 250 bp for the forward sequences and 200 bp for the reverse sequences. Once the "phyloseq" object was obtained with the corresponding taxonomic elimination, all sequences with less than two readings were eliminated to carry out the diversity analyses.

### Isolation of bacterial strains

Two grams of soil were suspended in 10 mL (final volume) of R2A culture medium [33] supplemented with 700 mM LiCl (Sigma-Aldrich-203637) and incubated 24 h at 28 °C with constant stirring (300 rpm). Subsequently, aliquots of 100 µL of this solution were used for growth on R2A agar plates. The plates were incubated at 28 °C during 24–48 h, and the colonies obtained were isolated.

### Minimal inhibitory concentration (MIC)

The minimal inhibitory concentration was determined using the protocol described by Elías et al., 2012 [34]. The initial solution contained LB medium supplemented with LiCl (4 M). Serial dilutions were set in 96-well microplates and inoculated with 5 µL from a previously grown bacterial culture. The plates were incubated at 28 °C, and their growth was evaluated after 24 h.

### Sulfide detection assay

The protocol used to evaluate H_2_S production was described by Shatalin et al. [35]. H_2_S production was evaluated in 5 mL of a bacterial culture grown in LB medium supplemented with 1, 1.5, and 2 mM cysteine. A paper soaked in lead acetate (100 mM) was attached under the cap. The tubes were incubated 24 h at 28 °C. Controls consisted of samples incubated without cysteine and/or without bacteria. H_2_S production was visualized by the change in the color of the papers obtained for each condition and quantified using the ImageJ software (http://imagej.nih.gov/ij/) considering a grayscale as described before [36].

### Biosynthesis of lithium sulfide nanoparticles

The method described for cadmium-sulfur nanoparticle biosynthesis developed by Monrás et al. [37] was used to evaluate the biosynthesis of lithium sulfide nanoparticles. Bacterial cultures were grown in LB medium until the stationary phase was reached, then the culture was centrifuged and the supernatant was discarded. The pellet was washed three times with distilled water, resuspended in borax-citrate buffer containing C_2_H_3_LiO_2_ (200 mM) and cysteine (2 mM), and incubated at 28 °C during 4, 16, and 24 h with constant stirring. Then, cultures were centrifuged 10 min at 7000 rpm, and the pellet was discarded. The supernatant containing the Li–S nanoparticles was filtered (0.22 µm filter) and used for subsequent purification steps.

### Metal sulfide detection protocol (auto metallography)

The biosynthesis of metal sulfide-containing nanomaterials was monitored using auto-metallography as has been described before [38–43]. The Silver Enhancer Kit, SE-100 (Sigma-Aldrich) was used for auto-metallography reaction using 100 µL biosynthesis reaction. Bacterial pellets containing metal sulfide nanoparticles were exposed to the silver enhancer solution for 10 min. Then, the mixture was centrifuged 2 min at 7000 rpm, and the silver enhancing solution was removed. Subsequently, a sodium thiosulfate solution was added for 3 min, and then the sample was observed in a microscope. Supernatants containing metal sulfide nanoparticles were exposed to the silver enhancer solution for 10 min. Then, a sodium thiosulfate solution was added, and after 3 min exposure the sample was observed in the microscope.

CdS and Li_2_S NPs used as controls in these experiments were chemically synthesized using a protocol previously described by our group [36]. Briefly, metal salts (cadmium chloride or lithium acetate), were incubated during 4 h at 90 ºC in presence of cysteine as sulfur donor (2 mM) and PBS buffer to produce metal sulfide NPs.

### Purification of nanoparticles

Extracellular nanoparticles were purified from cell supernatants following a previously described protocol [20, 44]. Supernatants containing NPs were filtered through 0.22 μm filters. Then, the NPs were concentrated in 10 kDa Amicon Tubes (Millipore). Finally, purified NPs were washed 10 times with distilled water.

### Transmission Electron Microscopy (TEM)

*P. rhodesiae* cells were grown under biosynthesis conditions (see above). Then, cells were concentrated by centrifugation, fixed with 2.5% glutaraldehyde, and infiltrated with epoxy resin. Cell Sects. (50–100 nm) were obtained using an ultramicrotome (EM UC 7, Leica Microsystems). Micrographs were collected using a Philips Tecnai 12 BioTwin microscope at 80 kV.

TEM micrographs of purified nanoparticles produced by *P. rhodesiae* were obtained using the same microscope. Then, the size of NPs was determined using the Pixelstick software (Plum Amazing Software LLC, Princeville, HI, USA) to establish a size-frequency histogram [45, 46]

### Lithium quantification on NPs

The presence of lithium was determined by spectrophotometry using the Thorin reagent (Sigma Aldrich) as described before [47, 48].

## Results

### Mineralogical characterization of Atacama salt flat soil sample

**Fig. 1:**
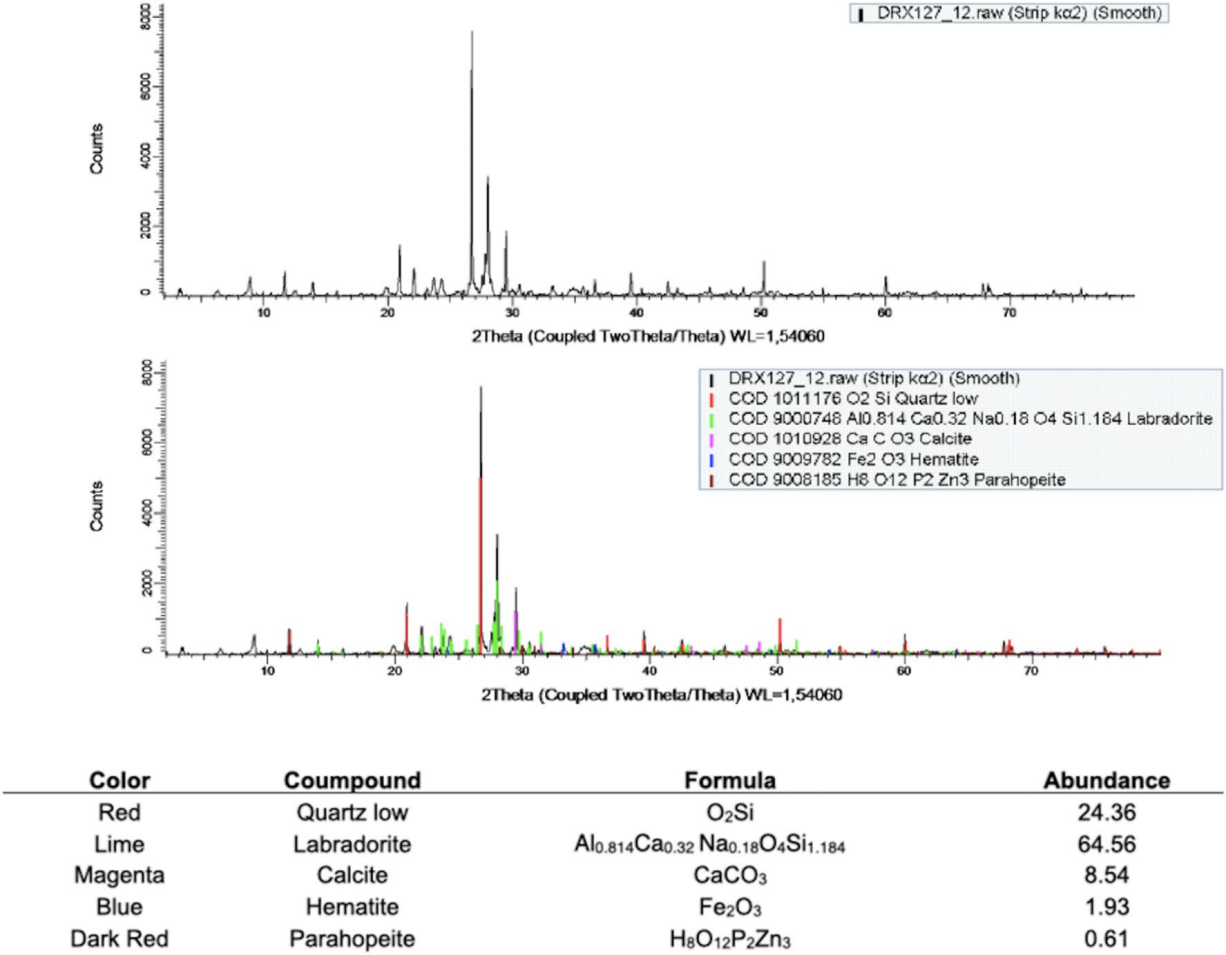
Mineral composition of the soil sample obtained from the Atacama Salt flat. XRD diffractogram of the sample indicating the compounds obtained and their proportion on the soil

The sample used for this research was obtained in a northern site of the Atacama salt flat. This zone is characterized by a high concentration of lithium (1570 ppm [49]) which is industrially extracted from brines [50–52]. The X-ray Diffraction (XRD) analysis of the soil revealed that the main minerals present are quartz, labradorite, calcite, hematite and scarce parahopeite (Fig. 1).

As has been previously reported, the geochemistry of the Atacama Desert surface is dominated by silica oxides (quartz, labradorite) [53, 55]. The presence of elements found in lower concentrations such as lithium, are not detected in these analyses given its low abundance in comparison with the oxides.

### Bacterial communities present in the Atacama salt flat soil sample

**Fig 2:**
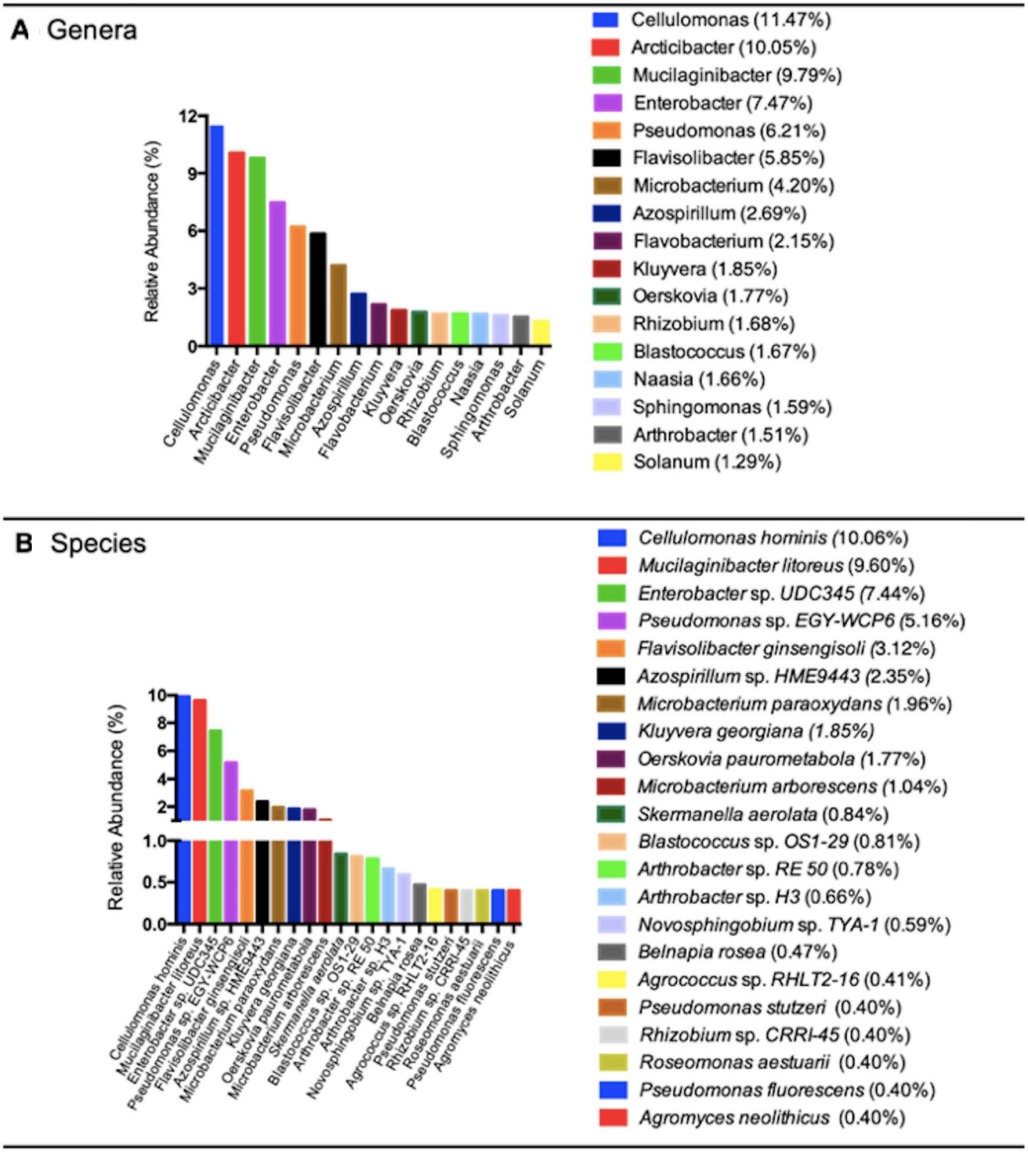
16 s metagenomic analysis of Atacama salt flat sample. Relative abundance of the most abundant bacterial genera and species present in the soil sample

A 16 s metagenomic analysis was performed on the soil sample to characterize the bacterial communities present. Figure 2 shows the relative abundances of the 17 most abundant genera (73% of the total) and the 22 most abundant species (51% of the total) present in the soil sample.

The analysis revealed that the most abundant bacterial genera in the sample were: *Cellulomonas* sp., *Arcticibacter* sp., *Mucilaginibacter litoreus*, *Pseudomonas* sp., and *Flavisolibacter* sp. As has been reported, the high salinity has generated an important selection pressure in this environment. Interestingly, microbial populations found in the different salt flats strongly differ, even at the family level. Significant differences in the composition of bacterial communities have been reported in salt flats with similar salinity levels and soil compositions [55, 56]. *Cellulomonas* sp. have been isolated from soils, and some species have been described as halotolerant [57, 58]. The Atacama Desert is an arid zone with extreme cold temperatures during the night, and extremely hot temperatures during the day, both conditions that could allow the development of bacterial genera such as *Arthrobacter* sp. and *Arcticibacter* sp., described in dry and cold environments in the Antarctic (Dry Valley) or Arctic [59–63].

### Isolation of lithium-resistant bacteria

With the purpose of isolating lithium-resistant bacteria present in the soil sample, the Atacama Desert soil was used to inoculate LB media supplemented with 500 mM LiCl. Twenty Li-resistant bacterial isolates were obtained after 48 h growth at 28 ºC. Subsequently, LiCl minimum inhibitory concentrations (MIC) were determined, and 3 isolates named D1N5.1, D2N2, and D2N5 were selected based on their high resistance to Li (Table 1). It should be noted that *E. coli* is not tolerant to LiCl and present a Minimal Inhibitory Concentration (MIC) of 200 mM [64]. Selected isolates were identified by the 16 s rRNA gene sequencing as *Pseudomonas rhodesiae, Planomicrobium koreense* and*, Pseudomonas* sp. with a 99, 88, and 92% identity respectly. The coverage percentage was 98% for *Pseudomonas rhodesiae,* 99% for *Planomicrobium koreense,* and 91% for *Pseudomonas sp.*

**Table 1:** Minimal Inhibitory Concentration (MIC) and sequence identity of lithium resistant isolates

Isolated	MIC LiCl (mM)	16 s rRNA—sequence identity	Accession number
D1N5.1	700	*Pseudomonas rhodesiae* (99%)	OM368334
D2N2	1000	*Planomicrobium koreense* (88%)	OM368354
D2N5	1000	*Pseudomonas* sp. (92%)	OM368357

### Sulfide production by lithium resistant isolates

**Fig. 3:**
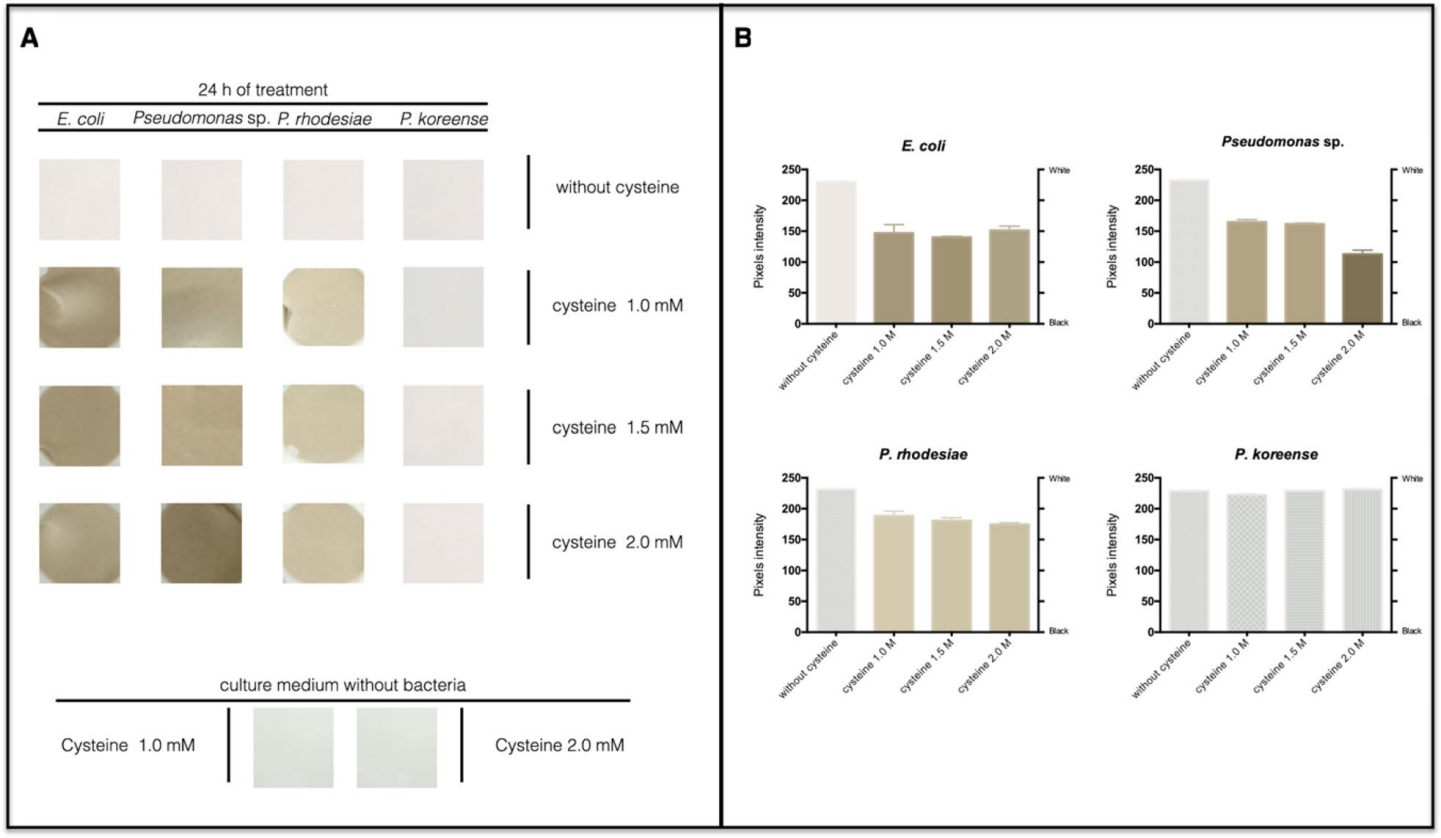
Sulfide production by lithium-resistant bacterial isolates. **A** The production of sulfide in the head spaces of bacterial cultures was determined as described previously [20, 24, 36, 63, 64]. *E. coli* was used as positive control for sulfide production in presence of cysteine [65]. **B** Pixels intensity of sulfide production by lithium-resistant bacteria and *E. coli.* Pixel intensity is inversely proportional to blackening of the image, with zero corresponding to black (no pixel intensity) and 255 to white (full pixel intensity)

In previous works we have described that H_2_S generation favors the bacterial biosynthesis of metal sulfides (MeS) nanoparticles [20, 24, 36, 65, 66]. Furthermore, methods to biosynthesize different MeS NPs involving the use of cysteine as S source for H_2_S production have been described to date. Based on this, we evaluated the ability of the three lithium resistant strains to produce H_2_S in the presence of cysteine. As shown in Fig. 3, *Pseudomonas rhodesiae* and *Pseudomonas* sp*.* produced high levels of sulfide in presence of cysteine. This result indicated that 2 mM cysteine is the ideal concentration for H_2_S production, and therefore we selected this concentration to evaluate the biosynthesis of lithium sulfide nanoparticles.

### Biosynthesis of lithium sulfide containing nanoparticles

The capability of lithium resistant selected strains to synthesize lithium sulfide nanomaterials was evaluated following the metal-sulfide detection protocol (see methods). Auto-metallography allows an easy and simple detection of different metal sulfide materials and has been previously used to detect metal-sulfide and gold nanoparticles [68–70]. This reaction involves a silver staining which in presence of metal-sulfide nanoparticles generates dark precipitates as a consequence of Ag binding, forming silver deposits that can be visualized by optical microscopy.

**Fig. 4:**
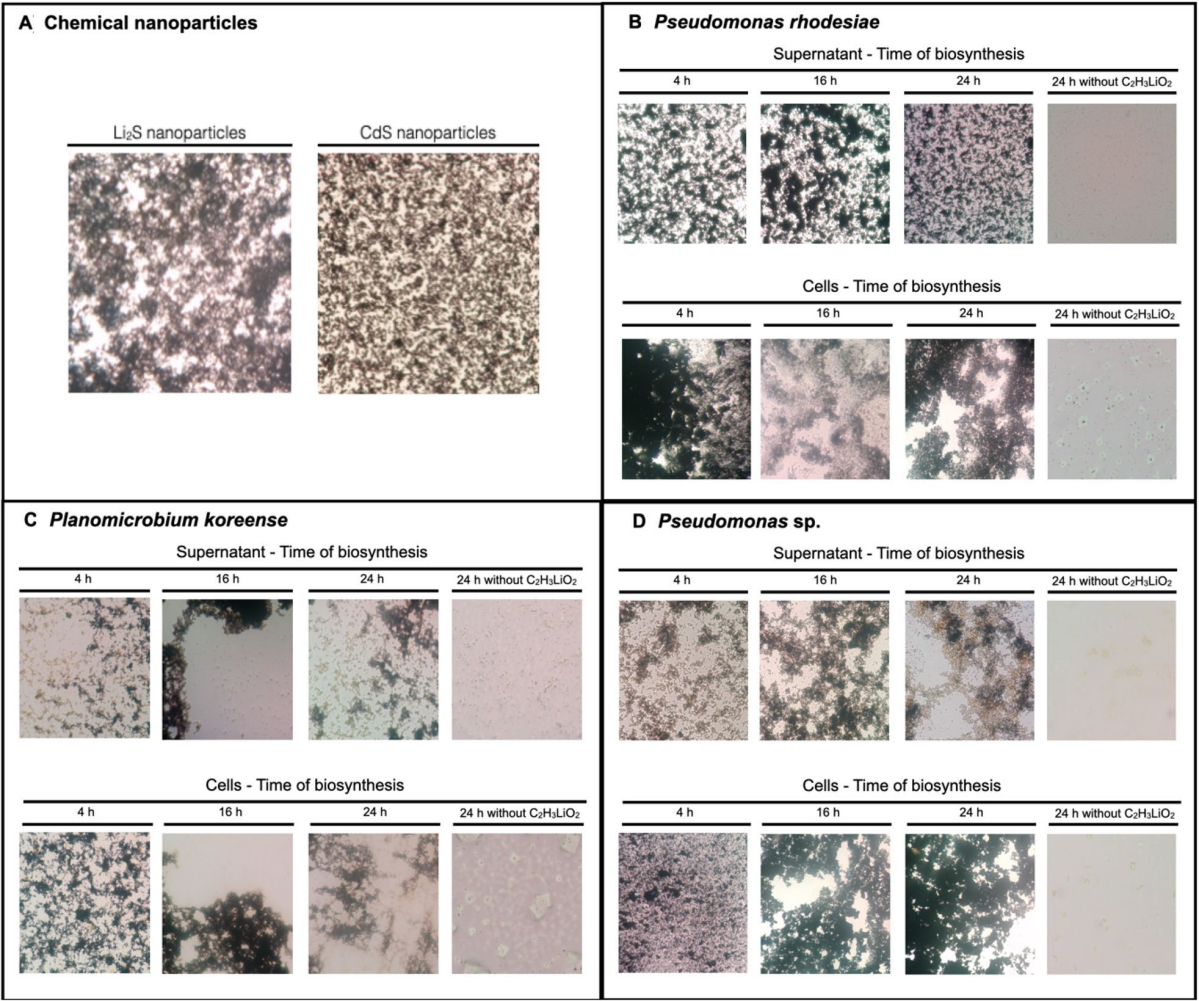
Kinetics of metal sulfide nanomaterials production by lithium-resistant isolates in presence of lithium and cysteine. **A** The auto-metallography reaction was evaluated on CdS and Li_2_S nanoparticles synthesized by chemical methods [36]. The production of metal sulfide nanomaterials was monitored in **B** *P. rhodesiae*, **C** *P. koreense,* and **D** *Pseudomonas* sp. at 4, 16, and 24 h as previously reported [38–43]. The intracellular and extracellular presence of lithium sulfide nanoparticles was evaluated

To date, this methodology has been used for the detection of different metal-sulfide NPs such as Cd, Pb, Zn, and Cu among others, however it has never been used for lithium-sulfide. Based on this, the detection of lithium-sulfide NPs was validated by using chemically synthesized nanoparticles as control (Fig. 4A).

As expected, lithium-sulfide nanoparticles synthesized by chemical methods gave a positive reaction in the auto-metallography reaction. Similar results were observed for CdS nanoparticles (Fig. 4A). Besides, no positive reaction was determined when the reaction was evaluated in presence of the precursors used for nanoparticle biosynthesis (not shown). Altogether, these experiments confirmed the specificity of auto-metallography for metal sulfide nanomaterials, including lithium-sulfide. The biosynthesis of lithium nanoparticles was evaluated in *P. rhodesiae* (D1N5.1), *P. koreensis* (D2N2), and *Pseudomonas* sp. (D2N5) isolates exposed to lithium and cysteine at different times (Fig. 4B–D).

No metal sulfide materials were detected by the auto-metallography reaction in cells and culture supernatants in absence of lithium acetate (Fig. 4B–D). On the other hand, dark precipitates revealing the presence of metal sulfide materials were observed in bacterial cells and culture supernatants of the three isolates when exposed to biosynthesis conditions at all times analyzed (4, 16 and 24 h). This result suggests that *P. rhodesiae* produce more intracellular and extracellular metal sulfide nanomaterials than the other isolates tested and based on this we decided to study the production of nanomaterials in this strain.

### Ultrathin sections of *P. rodhesiae* biosynthesizing lithium nanoparticles

**Fig. 5:**
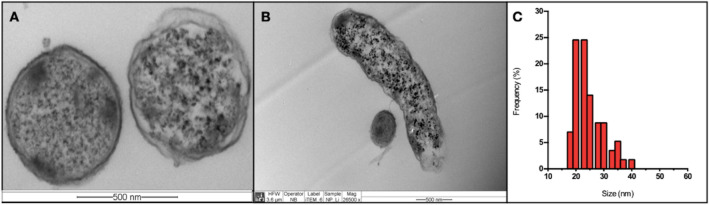
Transmission Electron Microscopy (TEM) of intracellular nanoparticles. **A**, **B** *Pseudomonas rhodesiae* under biosynthesis conditions. **C** Frequency size histogram of intracellular lithium nanoparticles from micrograph **B**

Ultrathin sections of *P. rodhesiae* cells exposed to biosynthesis conditions were prepared and analyzed by TEM to determine the presence and characteristics of nanomaterials inside cells. As expected, the presence of nanometric materials in the cytoplasm of *P. rodhesiae* cells was observed (Fig. 5A, B). The micrographs obtained allowed to establish a size-frequency histogram for intracellular Li–S nanoparticles, and sizes between 20 and 40 nm were determined (Fig. 5C). In addition, the micrographs revealed that biosynthesis conditions affect cell membranes, which could explain the presence of Li–S NPs in culture supernatants as determined in Fig. 4.

### Characterization of lithium nanoparticles produced by *P. rhodesiae*

Li–S nanoparticles biosynthesized by *P. rhodesiae* can be obtained in culture supernatants, a situation that favors their purification and subsequent characterization. Extracellular nanoparticles produced by *P. rhodesiae* were purified and the presence of lithium was determined by using the Thorin protocol [47, 48]. As expected, obtained results confirmed the presence of lithium on purified NPs revealing a content of lithium ~ 15%. Since the presence of proteins is a characteristic commonly reported on nanostructures produced by microorganisms, we determined the concentration of proteins on purified Li-NPs. Obtained results confirmed the presence of proteins on NPs produced by *P. rhodesiae* constituting ~ 1.1% of the nanostructure.

**Fig. 6:**
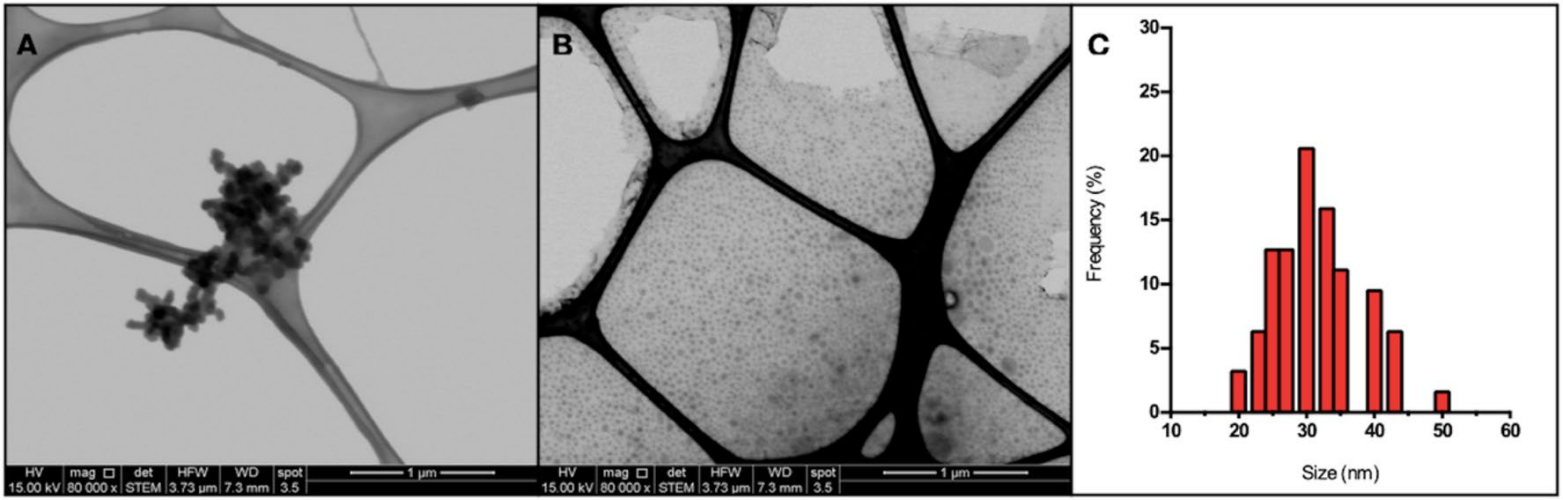
Transmission Electron Microscopy (TEM) of purified nanoparticles. **A**, **B** purified lithium nanoparticles and frequency size histogram of the nanoparticles. **C** Frequency size histogram of purified lithium nanoparticles from micrograph **B**

The size and morphology of purified Li-NPs biosynthesized by *P. rhodesiae* were characterized by Transmission Electron Microscopy (TEM). Circular nanoparticles with a size below 50 nm were determined (Fig. 6A). As has been previously observed for biologically produced nanomaterials, a fraction of Li–S nanomaterials tends to agglomerate. However, most of the purified nanoparticles are dispersed as shown in Fig. 6B. Micrographs obtained allowed to establish a size-frequency histogram for Li–S nanoparticles. Nanostructures with a size ranging from 20 to 50 nm (average ~ 30 nm) were observed (Fig. 6C), a result that agrees with the size observed for nanomaterials present inside *P. rhodesiae.* cell (Fig. 5C).

Additionally, the size of the purified lithium NPs was determined by Dynamic Light Scattering (DLS), indicating a hydrodynamic size of 44.62 nm in 80.2% of the sample (Additional file 1: Figure S1). This result agrees with size determinations described for lithium nanoparticles synthesized by chemical methods where sizes between 50 and 100 nm have been reported [71].

Altogether, results obtained in this work confirm the capability of microorganisms to biomineralize lithium salts and produce nanoparticles. In particular, the use of metal resistant bacteria isolated from extreme environments represents an interesting alternative to produce lithium sulfide nanomaterials using mild conditions of temperature, pH, and oxygen presence.

## Discussion

During the last decades, the production of lithium-ion batteries for electronic devices has strongly increased. As a consequence, there is great interest in developing more efficient forms of lithium batteries for energy storage. Lithium batteries based on the use of sulfurized lithium nanoparticles have emerged as a novel alternative because of the high energy storage capacity of nanoparticles (theoretical specific capacity of 1,166 mA h g^−1^) [72]. In general, Li–S nanomaterials are produced using chemical methods that involve high temperatures and inert atmospheres. No biological methods to produce lithium sulfide nanomaterials have been described to date. This is a relevant point for biological synthesis methods since one of the main difficulties in the synthesis of lithium nanoparticles is the destabilization that these nanostructures suffer when exposed to oxygen. This could be associated with the protein content determined in NPs and could explain why this type of nanoparticles tends to agglomerate, as was observed by TEM (Fig. 6). On the other hand, it would be interesting to evaluate this type of synthesis with anaerobic or facultative anaerobic microorganisms, which could be an alternative to increasing production volumes.

Nanoparticle biosynthesis using environmental microorganisms has allowed the production of new nanomaterials with unique properties. We recently reported the biosynthesis of stable fluorescent CdS nanoparticles at high NaCl concentrations by a cadmium-resistant bacterium isolated from the Dead Sea [20]. The biosynthesis method requires cysteine as a sulfur source and a cadmium salt (CdCl_2_). The cysteine is used to produce H_2_S, which diffuses through the cell membrane allowing the intra-and extracellular biosynthesis of nanoparticles [20, 36, 73, 74]. In this sense, extreme environments that present a high lithium concentration, such as the salt flats of northern Chile, represent ideal habitats for the selection of microorganisms with unique capacities to interact with this metal, tolerating high concentrations, and eventually biomineralize lithium in the form of lithium-sulfur nanomaterials.

The Atacama salt flat, located at 2300 m above sea level, presents unique environmental conditions such as high ultraviolet radiation, little annual rainfall, and extreme temperatures between day and night, which makes it an exciting place for bioprospecting [75–77]. In turn, the Atacama salt flat soils present a high concentration of lithium, with concentrations ranging 1570 ppm in some zones [49, 78, 79]. The soil sample used in this study was mainly composed by these minerals; quartz, labradorite, calcite, hematite and scarce parahopeite. Tapia, 2018 reported that the presence of silica oxides is correlated to aluminum oxides and, to a lesser extent, with calcium and iron oxides (calcite and hematite) in the Atacama Desert. Therefore, the surface of the Atacama salt flat soil sample agrees with the typical composition observed in desert environments. It is important to note that the selective pressure of this extreme habitat has been described as one of the most powerful described to date, mainly to its chaotropic environment [80, 81].

The bacterial genera with the highest abundance identified in the present study were *Cellulomonas*, *Arcticibacte*r, *Mucilaginibacter*, and *Pseudomonas*, among others. The literature has described that bacterium of the genus *Cellulomonas* have been isolated from arid high-altitude sites such as the Qinghai plateau in China [80], bacteria of the genera *Arcticibacter* and *Mucilaginibacter* have been isolated from soil samples of extreme cold sites, such as Svalbard in Norway and Antarctica [62, 83, 84]. The genus *Pseudomonas* corresponds to a ubiquitous microorganism, identified and isolated from various environments such as Antarctica, deserts, forests, seawater, and high-altitude sites [65, 85–88]. To date, reports on the microbial communities inhabiting lithium brines are scarce. Even the absence of bacteria has been reported in natural brines of the Atacama salt flat and Uyuni salt flat [89, 90]. In 2018, the presence of bacteria in the natural and concentrated brines of the Atacama salt flat was reported; this study indicated that the brines' bacterial abundance had marked differences, not establishing a correlation between the few communities present [78]. Halotolerant bacteria of the genera *Bacillus*, *Pseudomonas*, *Marinococcus*, *Vibrio*, among others, have been identified in soil samples from the Atacama salt flat [76, 89]. Halotolerant bacteria of the genera *Salinibacter*, *Pedobacter*, and *Alkalitalea* have been identified in soil samples from Uyuni salt flat [81].

Studies performed in a geographically close salt flat, such as the Gorbea salt flat in Chile, determined predominance of Gammaproteobacteria identifying *Enterobacter*, *Pantoea*, *Pseudomonas*, *Rhodanobacter*, *Shewanella*, and *Shigella* [56]. In addition, the analyses of these soils revealed high concentration of sulfate, identifying the presence of *Desulfomicrobium* and *Desulfosporosinu*s, which are sulfate-reducing bacteria that can use sulfite or thiosulfate as electron acceptors [92]. Authors also identified genes present in salt sediments associated with sulfur metabolism (sulfur production) such as *cysC, cysD, cysE, cysH, cysI, cysJ, cysM*, and *cysN*. Therefore, the microbiological exploration of the salt flats represents an excellent opportunity to discover new microorganisms capable of biosynthesizing sulfur metal nanoparticles, in particular nanoparticles composed of lithium and sulfur.

In this work, from saline sediments of the Atacama salt flat, we isolated lithium chloride resistant bacteria (30 and 40 g/L) with the ability to produce high concentrations of hydrogen sulfide in presence of cysteine. The lithium tolerance of the isolates is consistent with bacteria isolated from the Hombre Muerto salt flat, which is a geographically close salt flat in Argentina [55]. The 16 s rRNA sequencing determined that resistant isolates correspond to *Pseudomonas rodhesiae*, *Planomicrobium koreense*, and *Pseudomonas* sp*.* The ability of these isolates to biosynthesize Li–S nanomaterials was evaluated by auto-metallography analysis that indicated the presence of sulfur nanomaterials in the cells and the culture supernatant (Fig. 4). This phenomenon of extracellular biosynthesis has already been described for other biosynthesized nanoparticles. However, this is the first report describing the biological production of lithium-sulfur nanomaterials. The electron microscopy analysis of NPs biosynthesized revealed sizes consistent with lithium nanoparticles synthesized by chemical methods [71]. Although, to date, a mechanism for the biosynthesis of sulfurized metal nanoparticles has not been elucidated, some reports describe the activation of stress response pathways by cadmium during the biosynthesis of CdS nanoparticles, which implies the activation of genes related to damaged protein refolding and DNA repair proteins, such as MutS and DnaK [91]. Additionally, the generation of minicell-like structures has been observed at the poles of bacteria that produce metal sulfide nanoparticles [37, 65, 66, 94]. The production of minicells has recently been described to get rid of damaged proteins and thus decrease cell damage [95]. These studies could account for a method of exporting NPs from the intracellular medium through the cell poles. However, ultrathin sections of *P. rhodesiae* producing NPs indicated a homogeneous distribution of the nanoparticles inside the cell (Fig. 5). This phenomenon could be associated with lithium's lower toxicity in comparison with other metals such as cadmium and silver. The biomineralization process of this metal and lithium sulfide nanoparticles' formation would not be associated with a stress condition in cells.

## Conclusion

This work reports for the first time the biosynthesis of Li–S nanomaterials through the use of environmental bacteria. The biosynthesis method described in this work could be used to recover lithium from waste batteries and thus provide a solution to the accumulation of batteries [6, 96]. Additionally, this method will allow venturing into other salts as metal sources, such as lithium carbonate, which is the most common lithium precursor used in the industrial manufacture of rechargeable batteries [6, 7].

## Supplementary Information

**Additional file 1: Figure S1.** Dynamic Light Scattering (DLS) of lithium nanoparticles. Determination of hydrodynamic size of lithium nanoparticles biosynthesized by Pseudomonas rhodesiae.

## Data Availability

All data generated or analyzed during this study are included in this article and its additional information files.

## Abbreviations

TEM: Transmission electron microscopy
USGS: United States Geological Survey
NPs: Nanoparticles
GSH: Glutathione
CYS: Cysteine
MSA: Mercaptosuccinic Acid
DMS: Degrees, Minutes, Seconds in GPS coordinates
XRD: X-ray diffraction
LAS: Solid Analysis Laboratory at Universidad Andrés Bello
COD: Crystallography Open Database (COD)
LiCl: Lithium chloride
MIC: Minimal Inhibitory Concentration
H_2_S: Hydrogen sulfide
C_2_H_3_LiO_2_: Lithium acetate
MeS: Metal Sulfides
DLS: Dynamic Light Scattering (DLS)
CdCl_2_: Cadmium chloride
