# Supplementary material for: Biomineralization of lithium nanoparticles by Li-resistant Pseudomonas rodhesiae isolated from the Atacama salt flat
Source: Biol Res. 2022 Mar 16;55:12. doi: 10.1186/s40659-022-00382-6 (PMC8925236; doi:10.1186/s40659-022-00382-6)
Supplement: Supplementary file 1 — Additional file 1: Figure S1. Dynamic Light Scattering (DLS) of lithium nanoparticles. Determination of hydrodynamic size of lithium nanoparticles biosynthesized by Pseudomonas rhodesiae. [file 40659_2022_382_MOESM1_ESM.docx]

**Biomineralization of lithium nanoparticles by Li-resistant *Pseudomonas rodhesiae* isolated from the Atacama salt flat**

Bruna N.^1^, Galliani E.^1^, Oyarzún P.^2^, Bravo D.^3^, Fuentes F.^4^, Pérez-Donoso J.M.^1*^

^1^ BioNanotechnology and Microbiology Laboratory, Center for Bioinformatics and Integrative Biology (CBIB), Facultad de Ciencias de la Vida, Av. República # 330, Santiago, Chile.

^2^ Laboratorio de Análisis de Sólidos, Departamento de Ciencias Químicas, Facultad de Ciencias Exactas, Universidad Andrés Bello, Santiago, Chile.

^3^ Laboratorio de Microbiología Oral, Facultad de Odontología, Universidad de Chile, Santiago, Chile.

^4.^ Escuela de Geología, Facultad de Ciencias, Universidad Mayor, Av. Manuel Montt 367, Santiago, Chile .

* Correspondence: jose.perez@unab.cl

Supplementary Figure


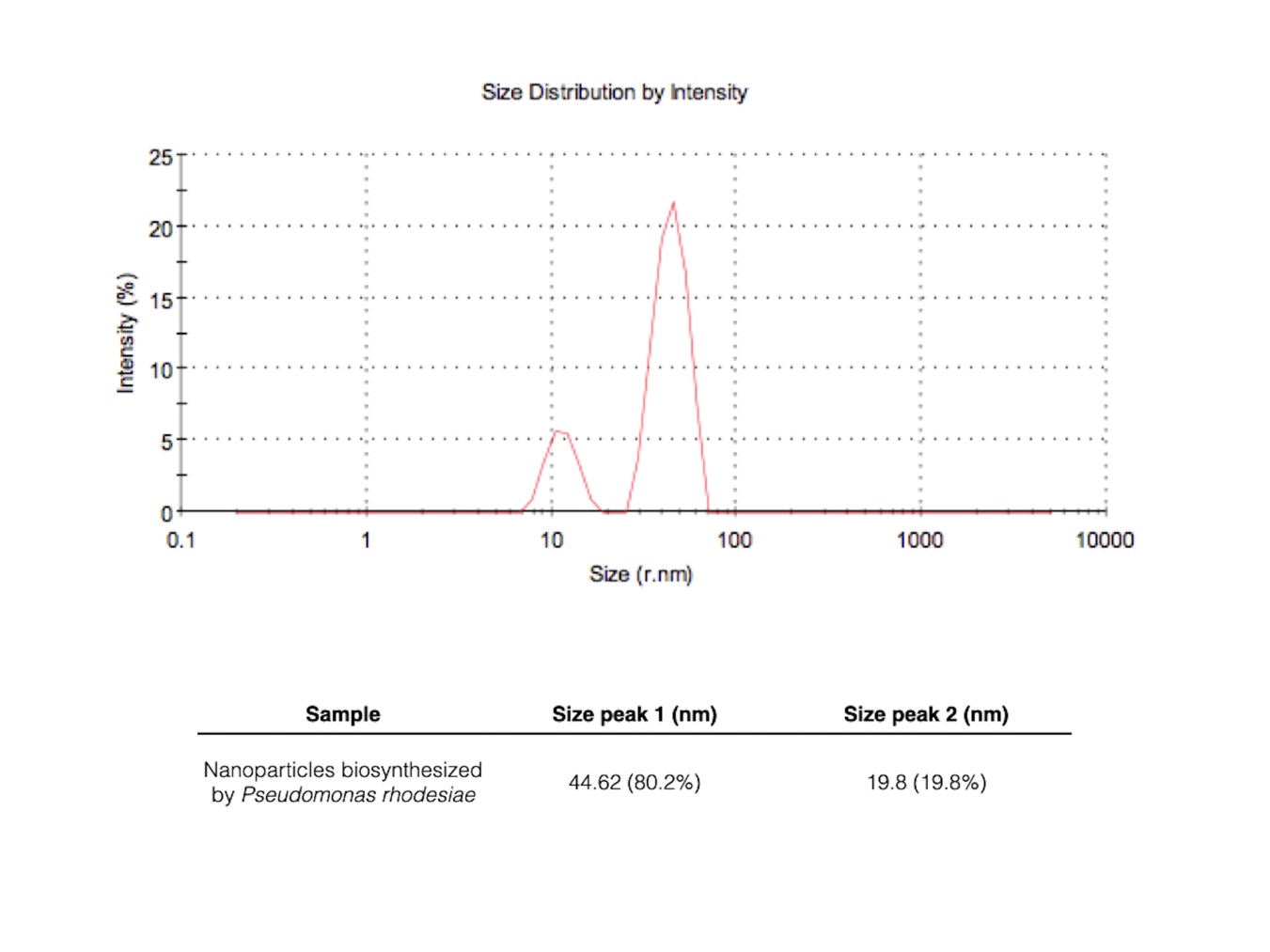


**Figure S1: Dynamic Light Scattering (DLS) of lithium nanoparticles**. Determination of hydrodynamic size of lithium nanoparticles biosynthesized by *Pseudomonas rhodesiae*.
